# Evaluating perioperative self-reported sleep quality in patients with a gynecologic malignancy

**DOI:** 10.1007/s00520-026-10493-5

**Published:** 2026-03-08

**Authors:** Ria M. Desai, Jason Silberman, Emma Reasner, Allison Grubbs, Karl Bilimoria, Emma L. Barber

**Affiliations:** 1 Northwestern University Feinberg School of Medicine, Chicago, IL USA; 2 Division of Gynecologic Oncology, Department of Obstetrics and Gynecology, Northwestern University Feinberg School of Medicine, Chicago, IL USA; 3 Division of Gynecologic Oncology, Department of Obstetrics and Gynecology, Rush University School of Medicine, Chicago, IL USA; 4 Surgical Outcomes and Quality Improvement Center (SOQIC), Department of Surgery, Indiana University School of Medicine, Indianapolis, USA

**Keywords:** Gynecologic malignancy, Sleep, Sleep quality, Perioperative

## Abstract

**Purpose:**

This study aims to evaluate patient-reported peri-operative sleep quality and identify demographic, clinical, and psychosocial factors associated with poor sleep perioperatively among individuals with gynecologic malignancies.

**Methods:**

This prospective study included patients with confirmed gynecologic malignancies who underwent surgery between September 2020 and October 2023 and were admitted to the hospital for at least one overnight stay. Preoperative sleep quality was assessed using the Pittsburgh Sleep Quality Index (PSQI), while postoperative sleep quality was measured with the Richards-Campbell Sleep Questionnaire (RCSQ). “Good” sleep was defined as a PSQI score < 5 or an RCSQ score > 50. Statistical analyses were conducted using t-tests, Pearson’s χ^2^, or Fisher’s exact tests, as appropriate.

**Results:**

The mean age of participants was 58.6 years (SD 13.2); 68.9% identified as white, 50% had ovarian cancer, and 54.6% had advanced-stage disease. Pre-operatively, 56.1% of patients reported poor sleep, most commonly due to overnight awakenings from nocturia (67.4%) and vasomotor symptoms (28%). Poor sleep was more common among patients with cervical or vulvar cancer (p = 0.02), those using sleep medications preoperatively (p = 0.002), and those with co-morbid anxiety and depression (p = 0.06). Good post-operative sleep on post-operative day 1 and on day of discharge was associated with increased use of opioid medications (p = 0.007 and 0.02, respectively). Overall, sleep quality significantly declined during hospitalization (p < 0.001).

**Conclusion:**

Poor perioperative sleep in patients with gynecologic cancers was linked to cancer type, mental health, pain management, and the hospital environment with sleep deteriorating during hospitalizations. Addressing these factors may offer meaningful opportunities to improve sleep.

## Introduction

Restful sleep is vital for overall health, functioning, and well-being. Impaired sleep can be characterized by disruptions in sleep duration, quality, and latency [1]. Such disruptions can have significant adverse effects on both physical and mental health. Sleep disturbances in adults have been linked to elevated cortisol levels, impaired glucose tolerance, and increased risk for coronary heart disease and mortality [2–6]. In addition to the physical consequences of poor sleep, disrupted sleep has been linked to heightened anxiety, reduced cognitive performance, and diminished emotional regulation [3, 6–8].

Postoperative sleep quality often deteriorates due to pain, anxiety, ambient light and noise, and frequent interruptions for patient assessments [9, 10]. Post-operative sleep disturbances in the hospital are associated with a decline in physical functioning, prolonged length of stay, and increased risk of re-hospitalization [11]. Poor post-operative sleep also amplifies pain perception, leading to greater analgesic use after discharge [1, 9]. Among older patients, in particular, poor sleep both before and after surgery increases the risk for post-operative delirium and mortality [1, 11, 12].

Sleep disturbances are particularly prevalent among cancer patients, affecting 30–93% of individuals in contrast to 9–33% in the general population [13]. Contributing factors may include pain, treatment side effects, psychological factors such as anxiety and depression, and tumor biology itself. In this population, poor sleep is associated with impaired functional status, worsened mental health, heightened pain, and quality of life [14]. Furthermore, women consistently report worse sleep quality and more disrupted sleep across all life stages due to neuroendocrine factors, thus placing patients with gynecologic cancers at increased risk for reduced sleep quality following surgery [15]. This study aims to examine patient-reported pre-operative sleep quality and to assess risk factors for poor pre-, and post-operative sleep amongst patients with gynecologic malignancies.

## Methods

This prospective cohort study evaluated sleep quality in patients admitted to the gynecologic oncology unit at Northwestern Memorial Hospital post-operatively. Patients were enrolled into the study between September 2020 to October 2023. This current study is a subgroup analysis evaluating the patient-reported sleep quality of patients with a histologically confirmed malignancy. Those who underwent surgery for benign disease were not included. This study was approved by the Northwestern University Institutional Review Board (STU0002144118).

Pre-operative sleep quality was assessed using the Pittsburgh Sleep Quality Index (PSQI), with instructions for patients to estimate their average sleep quality in the month leading up to their admission. This scale has been validated as a measure of longitudinal sleep quality in the outpatient setting, particularly in patients with chronic diseases such as cancer. Components of the PSQI include subjective sleep quality, sleep latency, sleep duration, habitual sleep efficiency, sleep disturbances, use of sleeping medication, and daytime dysfunction [16]. The PSQI ranges from 0 to 21, with higher scores indicating poorer sleep quality. For this study, a PSQI score of less than was 5 defined as “good sleep” [17]. Patients completed the PSQI during their pre-operative visit and were asked to estimate their average sleep quality over the prior month. Post-operative sleep quality was assessed using the Richards-Campbell Sleep Questionnaire (RCSQ), a tool consisting of five visual analog scales that measure sleep depth, latency, awakening frequency, and overall sleep quality [18]. This scale has been validated as a measure of sleep quality among admitted, acutely ill patients. Scores range from 0 to 100, with higher scores indicating better sleep quality. We defined good sleep as an RCSQ score greater than 50 [19]. Patients were asked to complete an RCSQ daily, beginning on post-operative day 1 and until their day of discharge. Note that differing scales were used to best capture longitudinal preoperative sleep and then short-term overnight sleep while patients were hospitalized postoperatively as different scales have been validated for these purposes.

Patient surveys were completed via REDCap using online questionnaires. These surveys collected self-reported information on demographic data (e.g. age, race, ethnicity), baseline pre-operative sleep quality, and post-operative sleep quality during admission. Additional demographic, clinical and surgical characteristics were abstracted from patient charts.

Baseline demographic and clinical characteristics of the study sample were reported using descriptive statistics. Patients were classified as having “good sleep” or “poor sleep” according to their survey responses as defined above and these groups were compared with respect to demographic, clinical and surgical characteristics. Continuous variables were analyzed using t-tests or Wilcoxon rank sum tests, while categorical variables were compared using Pearson’s χ^2^ or Fisher’s exact tests. Paired t-tests were conducted to compare patient-reported sleep scores over the duration of hospitalization. Patients that did not complete surveys on a given postoperative day were not included in analysis. All analyses were performed with Stata statistical software, version 18.0 (StataCorp).

## Results

The mean age of participants was 58.6 years (SD 13.2), with 91 (68.9%) of participants identifying as white, 24 (18.2%) identifying as black, 4 (3.0%) identifying as Asian, and 13 (9.9%) unreported. The most common cancer type in the cohort was ovarian (n = 66, 50%), followed by endometrial (n = 41, 31.1%), cervical (n = 12, 9.1%) and vulvar cancer (n = 9, 6.8%). Over half of the cohort was diagnosed with advanced stage disease (54.6%) and 72.0% of patients had open procedures (Table 1).

**Table 1:** Patient demographic and clinical characteristics

Characteristic	N = 132
**Age** Median (SD)	58.6 (13.2)
**Age Range** N (%)	
26–45	25 (18.9)
46–60	34 (25.8)
> 60	73 (55.3)
**BMI** Median (SD)	29.7 (8.6)
**BMI Range** N (%)	
< 18.5	3 (2.3)
18.5–24.9	38 (28.8)
25–29.9	39 (29.6)
> 30	52 (39.4)
**Race** N (%)	
White	91 (68.9)
Black	24 (18.2)
Asian	4 (3.0)
Not Reported	13 (9.9)
**Cancer Type** N (%)	
Uterine	41 (31.1)
Ovarian	66 (50.0)
Cervical	12 (9.1)
Vulvar	9 (6.8)
Other	4 (3.0)
**Stage** N (%)	
I	50 (37.9)
II	8 (6.1)
III	51 (38.6)
IV	21 (15.9)
Unknown	2 (1.5)
**Surgical Route** N (%)	
Minimally Invasive	31 (23.5)
Open	95 (72.0)
Other	6 (4.5)
**Charlson Comorbidity Index** N (%)	
0–2	28 (21.2)
3–5	20 (15.2)
6 +	84 (63.6)
**Length of Stay** Median (IQR)	3 (1–5)
**Sleep Medication use Pre-Operatively,** N (%)	
Yes	18 (13.6)
No	114 (86.4)
**Anxiety/Depression** N (%)	
Yes	36 (27.3)
No	96 (72.7)

Pre-operatively, patients reported a median time to fall asleep of 15 min (IQR 5–30), with an average of 6.7 h (SD 1.6) of sleep per night. According to composite PSQI scores, a total of 58 patients (43.9%) reported good pre-operative sleep, while 74 patients (56.1%) reported poor sleep. Most patients reported overnight awakenings (59.1%) and the need to use the bathroom overnight (67.4%) at least three times per week, with an additional 18.9% and 16.7% reporting these issues at least once a week, respectively. Over 28% of patients reported issues with feeling hot at night at least once a week, with 11.4% reporting this issue to occur at least three times per week. Other factors that led to at least weekly nighttime disturbances included pain (25.8%), feeling hot (28.1%) or cold (15.9%), coughing or snoring (10.6%), nightmares (6.8%), or trouble breathing (4.6%) (Table 2).

**Table 2:** Baseline Pittsburgh Sleep Quality Index (PSQI) results

**Time to fall asleep, minutes** (median, IQR)	15 (5–30)
**Hours of sleep per night** (mean, SD)	6.7 (1.6)
**Overnight Awakenings** N (%)	
Not during the past month	21 (15.9)
Less than once a week	8 (6.1)
Once or twice a week	25 (18.9)
Three or more times a week	78 (59.1)
**Bathroom Overnight** N (%)	
Not during the past month	12 (9.1)
Less than once a week	9 (6.8)
Once or twice a week	22 (16.7)
Three or more times a week	89 (67.4)
**Trouble Breathing** N (%)	
Not during the past month	5 (3.8)
Less than once a week	121 (91.7)
Once or twice a week	5 (3.8)
Three or more times a week	1 (0.8)
**Coughing/Snoring** N (%)	
Not during the past month	104 (78.8)
Less than once a week	14 (10.6)
Once or twice a week	7 (5.3)
Three or more times a week	7 (5.3)
**Cold at Night** N (%)	
Not during the past month	87 (65.9)
Less than once a week	24 (18.2)
Once or twice a week	9 (6.8)
Three or more times a week	12 (9.1)
**Hot at Night** N (%)	
Not during the past month	75 (56.8)
Less than once a week	20 (15.2)
Once or twice a week	22 (16.7)
Three or more times a week	15 (11.4)
**Nightmares** N (%)	
Not during the past month	101 (76.5)
Less than once a week	22 (16.7)
Once or twice a week	4 (3.0)
Three or more times a week	5 (3.8)
**Pain at Night** N (%)	
Not during the past month	77 (58.3)
Less than once a week	21 (15.9)
Once or twice a week	13 (9.9)
Three or more times a week	21 (15.9)
**Other Reasons for Awakening** N (%)	
Not during the past month	90 (68.2)
Less than once a week	3 (2.3)
Once or twice a week	12 (9.1)
Three or more times a week	15 (11.4)
Unknown	12 (9.1)

Patients reporting good sleep had similar baseline characteristics to those reporting poor sleep regarding age, BMI, race, and stage (Table 3). There were notable differences in cancer type (p = 0.02) amongst sleep quality groups, with ovarian and uterine cancer being more prevalent in good sleep (56.9%, 34.5%, respectively) versus poor sleep (44.6%, 28.4%, respectively). A higher proportion of patients with poor sleep had cervical (12.2%) or vulvar cancer (12.2%) compared to those with good sleep (5.2% cervical and 0% vulvar). Additionally, patients reporting poor sleep had a trend toward higher rates of anxiety or depression (33.8% vs 19.0%, p = 0.06) and had significantly increased use of sleep medications pre-operatively (21.6% vs 3.5%, p = 0.002). There was no difference in observed length of stay nor 30-day postoperative complication rate amongst pre-operative sleep quality groups (Table 3).

**Table 3:** Comparison of pre-operative pre-operative sleep quality (PSQI) groups

**Variable**	**Good Sleep** (%)	**Poor Sleep** (%)	**p-value**
Number	58 (43.9)	74 (56.1)	
Age (Mean, SD)	58.5 (12.1)	58.8 (14.0)	0.91
BMI (Mean, SD)	30.1 (9.0)	29.5 (8.2)	0.70
Length of Stay (Median, IQR)	3 (2–4)	3 (1–5)	0.80
Race White Black/African American Asian Not reported	42 (72.4) 11 (19.0) 2 (3.5) 3 (5.2)	49 (66.2) 13 (17.6) 2 (2.7) 10 (13.5)	0.463*
Cancer Type Uterine Ovarian Cervical Vulvar Other	20 (34.5) 33 (56.9) 3 (5.2) 0 (0) 2 (3.5)	21 (28.4) 33 (44.6) 9 (12.2) 9 (12.2) 2 (2.7)	0.02*
Stage Stage I Stage II Stage III Stage IV Unknown	17 (29.3) 3 (5.2) 23 (39.7) 13 (22.4) 2 (3.5)	33 (44.6) 6 (6.8) 28 (37.8) 8 (20.8) 0 (0)	0.11*
Charlson Comorbidity Index 0–2 3–5 6 +	11 (19.0) 7 (12.0) 40 (69.0)	17 (23.0) 13 (17.6) 44 (59.4)	0.53*
Sleep Meds Pre Op Yes No	2 (3.5) 56 (96.5)	16 (21.6) 58 (78.4)	0.002*
Anxiety/Depression Yes No	11 (19.0) 47 (81.0)	25 (33.8) 49 (66.2)	0.06
Complications No Yes	41 (70.7) 17 (29.3)	43 (58.1) 31 (41.9)	0.14

^*^Fisher's Exact test

**Fig. 1:**
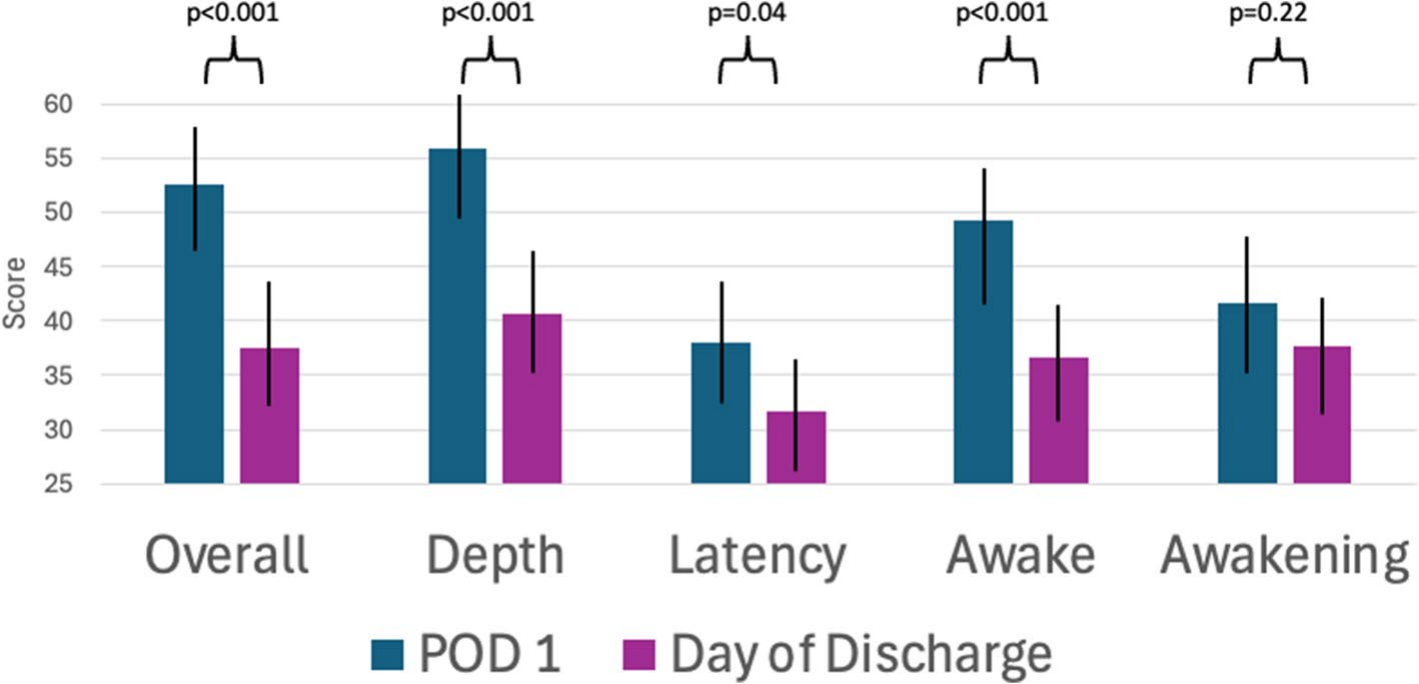
Comparison of mean Richards-Campbell Sleep Questionnaire (RCSQ) component scores from post-operative day 1 (POD1) to the day of discharge

Post-operative sleep scores worsened over the duration of hospitalization (Fig. 1). This included significant worsening in patient-reported sleep depth (p < 0.001), time to fall asleep (p = 0.04), ability to return to sleep when awakened (p < 0.001), and sleep quality overall (p < 0.001). Patients reporting good sleep on post-operative day 1 were more commonly those with ovarian cancer (61.8% vs 39.0%), while patients with poor sleep were more commonly patients with uterine cancer (40.7% vs 19.1%, p = 0.02). Route of surgery was also significantly different between groups, with a higher proportion of patients reporting good sleep having undergone open surgery (85.3% vs 61.0%, p = 0.006). Comparisons of morphine milligram equivalents (MME) demonstrated higher mean MME use on post-operative day 1 (POD1) amongst patients reporting good sleep, compared to patients reporting poor sleep (26.1 vs 13.2, p = 0.007). This statistically significant difference persisted between patients on the day of discharge (16.6 vs 7.2, p = 0.02) (Table 4). Pre-operative sleep quality was not associated with postoperative sleep quality with no association between good pre-operative sleep and good post-operative sleep, either on POD1 or day of discharge.

**Table 4:** Comparison of post-operative Richards-Campbell Sleep Questionnaire (RCSQ) groups on post-operative day 1 and on the day of discharge

	Post-Operative Day 1			Day of Discharge		
Variable	Poor Sleep N (%)	Good Sleep	P-Value	Poor Sleep N (%)	Good Sleep	P-Value
Overall	59 (46.5)	68 (53.5)		78 (69.0)	35 (31.0)	
Pre-Operative Sleep Quality Poor Sleep Good Sleep	35 (50.7) 24 (41.4)	34 (49.3) 34 (58.6)	0.29	40 (67.8) 38 (70.4)	19 (32.2) 16 (29.6)	0.77
Race White Black Asian Not Reported	40 (67.8) 10 (17.0) 2 (3.4) 7 (11.9)	47 (69.1) 13 (19.1) 2 (2.9) 6 (8.8)	0.96	58 (74.4) 10 (12.8) 1 (1.3) 9 (11.5)	25 (71.4) 8 (22.9) 1 (2.9) 1 (2.9)	0.23
Age Category 26–45 46–60 > 60	8 (13.6) 14 (23.7) 37 (62.7)	17 (25.0) 19 (27.9) 32 (47.1)	0.15	14 (18.0) 22 (28.2) 42 (53.9)	8 (22.9) 10 (28.6) 17 (48.6)	0.81
BMI category < 18.5 18.5–24.9 25–29.9 > 30	2 (3.4) 18 (30.5) 15 (25.4) 24 (40.7)	1 (1.5) 18 (27.5) 23 (33.8) 26 (38.2)	0.69	2 (2.6) 19 (24.4) 26 (33.3) 31 (39.7)	0 (0) 11 (31.4) 10 (28.6) 14 (40.0)	0.68
Anxiety/Depression No Yes	47 (79.7) 12 (20.3)	46 (67.7) 22 (32.3)	0.13	60 (76.9) 18 (23.1)	23 (65.7) 12 (34.3)	0.21
Cancer Type Uterine Ovarian Cervical Vulvar Other	24 (40.7) 22 (39.0) 4 (6.8) 5 (8.5) 3 (5.1)	13 (19.1) 42 (61.8) 8 (11.8) 4 (5.9) 1 (1.5)	0.02	26 (32.3) 36 (46.2) 7 (9.0) 6 (7.7) 3 (3.9)	8 (22.9) 18 (54.3) 4 (11.4) 3 (8.6) 1 (2.9)	0.83
Stage Stage I Stage II Stage III Stage IV Unknown	23 (39.0) 2 (3.4) 24 (40.7) 8 (13.6) 2 (3.4)	23 (33.8) 6 (8.8) 27 (39.7) 12 (17.7) 0 (0)	0.42	32 (40.0) 2 (2.6) 32 (41.0) 11 (14.1) 1 (1.3)	10 (28.6) 6 (17.1) 12 (34.3) 7 (20.0) 0 (0)	0.05
Route MIS Open Other	18 (30.5) 36 (61.0) 5 (8.5)	9 (13.2) 59 (85.3) 1 (1.5)	0.006	19 (24.4) 56 (71.8) 3 (3.8)	8 (22.9) 25 (71.4) 2 (5.7)	0.90
Sleep Meds Preop No Yes	52 (88.1) 7 (11.9)	59 (86.8) 9 (13.2)	0.82	69 (88.5) 9 (11.5)	29 (82.9) 6 (17.1)	0.42
Morphine use (mean, 95% CI)	13.2 (8.1–18.3)	26.1 (18.5–33.7)	0.007	7.2 (4.0–10.5)	16.6 (6.6–26.6)	0.02

## Discussion

In our study evaluating patient-reported peri-operative sleep quality, we identified various clinical and patient factors associated with poor sleep. Regarding pre-operative sleep quality, we found that patients reporting good sleep more commonly had ovarian and uterine cancers, while patients with vulvar and cervical cancers more commonly reported poor pre-operative sleep. Additionally, patients with anxiety and depression exhibited a trend toward poor pre-operative sleep, though this did not reach statistical significance. The bidirectional relationship between mood disorders and sleep disturbances is well established in both the general population and in individuals living with chronic illnesses [20–24]. This may be particularly relevant for women with cervical and vulvar cancer, who often experience heightened levels of psychological distress, potentially contributing to poorer sleep quality [25–27].

Within the PSQI, several factors significantly contributed to poor sleep. The most commonly reported reason for overnight awakenings was the need to use the bathroom. Nocturia, which increases with age, has been linked to poor sleep quality, worsening depression, and mobility issues—factors that can negatively impact post-operative recovery [28–30]. Additionally, more than 28% of patients reported experiencing feeling hot at night. Given the average age of 59 in our cohort, these symptoms were likely due to vasomotor symptoms of menopause (VSM) [31, 32]. Improving screening for nocturia and VSM should be considered, as targeted interventions could alleviate these symptoms and, in turn, enhance sleep quality.

Post-operatively, several correlated factors had associations with good sleep. Chief among these was morphine use, with patients reporting good subjective sleep having received higher levels of MME, both on post-operative day 1 (p = 0.007) and on the day of discharge (p = 0.02). Relatedly, patients with ovarian cancer and those who underwent open surgery, reported better sleep, likely due to the higher levels of MME prescribed for open surgery patients [33]. The relationship between sleep and postoperative pain is complex and an ongoing area of investigation [34]. While poor sleep and pain exacerbation likely have a bidirectional relationship, our data suggests that pain management with opioids may help to improve sleep in the acute post-operative setting. It is important to consider that the sleep measurements discussed here are subjective in nature, and while patients may report improved sleep, opioids can interfere with normal sleep architecture. Therefore, prior to developing conclusions about the relationship between opioids and sleep, further studies should evaluate the relationship between opioid usage and objective sleep measures in the post-operative setting, which may demonstrate a more complex relationship. However, the potential for dangerous side effects with excessive opioid use underscores the importance of multi-modal pain interventions. All patients enrolled in this study were also managed on our institutional Enhanced Recovery After Surgery (ERAS). These protocols may help to reduce opioid burden while optimizing pain control [35, 36].

Notably, we found that patient-reported sleep quality deteriorated over the course of the hospital stay. This decline may, in part, be attributed to ambient disturbances such as light, noise, and frequent disturbances [9, 10]. Additionally, several important confounding factors must be considered when interpreting this finding. Patients who experience post-operative complications—such as infections, the need for blood transfusions, or increased clinical monitoring—are more likely to have frequent sleep interruptions during hospitalization. These same factors may also independently contribute to prolonged length of stay, making it difficult to disentangle the relationship between poor sleep and extended hospitalization. In order to address some of the factors that may impact sleep, quality improvement initiatives aimed at enhancing sleep quality in hospitals are becoming more prevalent, particularly as the healthcare system shifts toward quality-based care. Surveys, such as HCAHPS, specifically assess hospitals’ performance in providing a quiet environment to support patient rest at night, highlighting the importance of sleep quality as an outcome of interest for health systems [37–40].

This study has several limitations worth noting. The small sample size may have limited our ability to detect meaningful associations and perform more complex multivariate analysis, underscoring the need for future studies with larger populations. Additionally, the sample was recruited from a single hospital system, which may limit generalizability of our findings. The voluntary and subjective nature of the self-reported data introduces the potential for participation bias. There were also limitations in assessing the association between pre- and post-operative sleep quality given the differences in questions and scoring between the PSQI and RCSQ. Two scales were necessary to obtain two different types of sleep quality data, longitudinal preoperatively versus acute hospital-related sleep quality. Because of this, direct comparisons using the same instruments cannot be made between sleep in these two periods. Despite these limitations, the current study highlights meaningful differences in sleep quality related to cancer type, co-morbidities and post-operative pain management, along with a notable trend of deteriorating sleep quality during hospital stays. Future work should provide interdisciplinary targeted interventions to improve sleep quality among patients with gynecologic malignancies undergoing surgery.

## Data Availability

No publicly available dataset was used for this study. Study data is not shared publicly to protect participant privacy.
